# Limbic and cortical control of phonation for speech in response to a public speech preparation stressor

**DOI:** 10.1007/s11682-019-00102-x

**Published:** 2019-05-02

**Authors:** Maria Dietrich, Richard D. Andreatta, Yang Jiang, Joseph C. Stemple

**Affiliations:** 1 Department of Speech, Language and Hearing Sciences, University of Missouri, 308 Lewis Hall, Columbia, MO 65211 USA; 2 Department of Communication Sciences and Disorders, University of Kentucky, 120 Wethington Bldg, 900 S. Limestone, Lexington, KY 40536 USA; 3 Department of Behavioral Science, University of Kentucky, 113 Medical Behavioral Science Building, Lexington, KY 40536 USA

**Keywords:** Functional MRI, Limbic, Stress, Larynx, Voice disorders, Muscle tension dysphonia

## Abstract

Knowledge on brain networks subserving vocalization in vocally healthy individuals under various task conditions is scarce but paramount to understand voice disorders. The aims of our study were to determine (1) the effect of social-evaluative stress on the central neural control of phonation underlying speech production; and (2) the neural signature, personality profile, and aerodynamic vocal function in relation to salivary cortisol responses. Thirteen vocally healthy females underwent an event-related sparse-sampling fMRI protocol consisting of voiced and whispered sentence productions with and without exposure to the social-evaluative stressor public speaking anticipation. Participants completed a personality questionnaire, rating scales of negative emotional state, and provided salivary cortisol samples. In the total sample, the task contrast of voiced productions revealed that stressor exposure resulted in a peak activation in the right caudate with concomitant deactivations in the bilateral pgACC and aMCC, and right IFG, BA 9, BA 10, insula, putamen, and thalamus. There were individual differences in stressor-induced brain activations as a function of stress reactivity with greater cortisol reactivity linked with lower laryngeal motor cortex activity and lower scores on aspects of extraversion. Our data confirm that stress alters the phonatory control for speech production through limbic-motor interactions. The findings support the Trait Theory of Voice Disorders (Roy and Bless 2000) and help provide critical insights to the study of voice disorders such as primary muscle tension dysphonia.

## Introduction

Phonatory control for speech operates under fluctuating cognitive and emotional states. While humans exert volitional control over phonation underlying speech production via primarily direct corticomotoneuronal pathways (Jürgens 2002), the direct or indirect impact of emotion on phonation for speech remains ambiguous at best. Primary vocal motor areas include the laryngeal motor cortex (LMC), premotor cortex, supplementary motor area (SMA), and lobule VI of the cerebellum, whereas secondary areas include the cingulate motor area, the ventral tier nuclei of the thalamus, the putamen, frontal operculum, and the anterior insula (Brown et al. 2009). In contrast to humans, primates regulate vocalizations through a limbic vocal motor pathway that relies on the anterior cingulate cortex (ACC) and periaqueductal gray (PAG) (Jürgens 2002). In humans, the analogous limbic vocal motor pathway plays a role in non-speech emotional vocalizations, such as crying or laughing (Ludlow 2005). While limbic activations have been reported during vocalizations for the purpose of speech (Haslinger et al. 2005; Loucks et al. 2007; Olthoff et al. 2008; Schulz et al. 2005), it has not been unequivocally determined whether these are epiphenomenal or essential to human phonatory control and speech production (Ludlow et al. 2008). A deeper understanding of the contribution of limbic activity during voice production such as the effects of emotion and psychological stress is warranted given the potentially profound modulatory effects of voice-related sensorimotor processing that may help to explain the origins of selected classes of voice disorders (Dietrich et al. 2012).

The *Trait Theory of Voice Disorders* (Roy and Bless 2000) has been proposed as a means for describing the potential clinical relevance of the limbic system and its interference role with voice production. The central hypothesis is that functional pathways between the limbic system and laryngeal sensorimotor control regions may help to explain individual differences in dispositional-related laryngeal motor behavior and the risk for voice disorders. For example, a predisposition to react with behavioral inhibition to punishment, novelty, or threat, assumed to be greater in individuals with introverted than extroverted traits, would interrupt or halt motor cortical activity and result in heightened and/or disorganized peripheral laryngeal muscle activity. Such heightened and/or disorganized muscle activity is thought to underlie complaints of perceived vocal effort and strain, which are, coincidently, hallmark symptoms of primary muscle tension dysphonia.

Data from connectivity analyses for the LMC may be compatible with the *Trait Theory.* Structural connectivity between the LMC and the middle cingulate cortex (MCC) has been shown and to a lesser extent connectivity with the dorsolateral prefrontal cortex (dlPFC) and midbrain (Simonyan et al. 2009). Additionally, functional connectivity data for the LMC have indicated that connectivity with the ACC and MCC was negative (weakened) during syllable productions, but positive connectivity would be plausible for complex speech, and that positive (enhanced) connections were found for the dlPFC, ventrolateral PFC (vlPFC), and midbrain. In the limbic system, the ACC integrates emotional and motivational states into cognitive processes, with its output influencing motor control for voice or vocal control (Paus 2001; Paus et al. 1993). Further, the ACC, amygdala, and septo-hippocampal system are neural correlates of behavioral inhibition (McNaughton and Corr 2004).

The clinical implications for this study are that in some patients with voice disorders, acute or chronic life stress accompanies the occurrence of primary muscle tension dysphonia, a voice disorder in the absence of vocal fold lesions, vocal fold paralysis, or a diagnosed psychological disorder (Dietrich et al. 2008; Roy 2003; Verdolini et al. 2006). In contrast, secondary muscle tension dysphonia occurs when vocal fold lesions or neurological or psychological etiologies are the primary source of the voice disorder and lead to compensatory muscular responses (Verdolini et al. 2006). The limbic system is involved in the initial processing of information and regulation of the stress response (Dedovic et al. 2009b) and participates in vocal control. Consequently, studying the relation between stress and the limbic and cortical control of phonation for speech production is a promising avenue to improve our understanding of individual differences in vocal control and the ever-present risks for the emergence of stress-related voice disorders (Dietrich et al. 2012).

Only three studies have been completed to date that explored the neurobiological bases of primary muscle tension dysphonia; (1) a cross-sectional study (Kryshtopava et al. 2017), (2) a case study pre- and post-successful laryngeal reposturing treatment (Roy et al. 2017), and (3) a study that explored two patients with muscle tension aphonia pre- and post-successful circumlaryngeal therapy (Spengler et al. 2017). The case studies, in particular, supported the hypothesis that dysfunctional limbic-motor interactions may underlie muscle tension laryngeal dysphonias, consistent with the *Trait Theory*. Still absent in the literature is detailed knowledge on cortical and subcortical brain networks subserving vocalization under various task conditions in vocally healthy individuals (Ludlow 2005; Simonyan et al. 2009). As such, we are lacking studies that have collected both physiological and brain responses to stress and individual reactivity during overt and covert voice production. This particular research direction, informed by the *Trait Theory*, is described in the *Psychobiological Framework for Studying Psychological Stress and its Relation to Voice Disorders* (Dietrich and Verdolini Abbott 2008; Helou 2014). This framework has been successfully used to study peripheral vocal function during a stress reactivity protocol (Dietrich and Verdolini Abbott 2012, 2014; Helou 2014). Integrating stress reactivity perturbations and fMRI protocols to study voice and speech production is novel and necessitates a feasibility and pilot study. Our central aim in this report was to provide initial data that imposed stress alters phonatory control for speech, which would support a key claim of the *Trait Theory* that individuals who score low on extroversion are prone to interrupt or halt motor cortical activity in response to novelty or threat with further implications for peripheral laryngeal function.

The objectives of our feasibility and pilot study were to determine (1) the effect of social-evaluative stress on the central neural control of phonation underlying speech production; and (2) the neural signature, personality profile, and aerodynamic vocal function of individuals in relation to their salivary cortisol responses. Based on the *Trait Theory*, we hypothesized that stressor exposure will alter laryngeal motor and premotor control, especially in those individuals who are stress responders. Based on previous fMRI research using a “speech preparation” activity as a stressor (Lorberbaum et al. 2004; Wager et al. 2009) and the Montreal Stress Imaging Task (Pruessner et al. 2008), we expected the following stressor-induced neural events: activations in the primary motor cortex (M1), premotor cortex, pre-SMA, middle frontal gyrus (MFG), ACC, MCC, insula, HC, thalamus, caudate, PAG, and cerebellum; deactivations in the sensorimotor cortex (S1, M1), ventromedial PFC (vmPFC), OFC, vlPFC, posterior cingulate cortex (PCC), superior temporal gyrus (STG), insula, putamen, amygdala, HC, and cerebellum. Furthermore, we expected that individuals who would be more stress reactive based on cortisol would be characterized by lower self-esteem (Dickerson and Kemeny 2004), lower extroversion (Dietrich and Verdolini Abbott 2014), and greater laryngeal airway resistance than the non-responder group. Greater laryngeal airway resistance is a proxy measure of increased muscle tension in the laryngeal system of vocalizing humans (Hillman et al. 1989).

## Materials and methods

### Participants

Participants in the study were 13 females with a mean age of 21.7 years (*SD* = 4.6, range: 18–35 years). All participants were right-handed, native speakers of English, and in good physical, mental, and vocal health. Considering that this was a feasibility and pilot study, a pre-screening protocol was used to pre-organize the participant pool on the personality trait of stress reactivity. The goal was to balance the number of participants who scored below and above the norm on the 12-item true/false *Stress Reaction* scale from the Multidimensional Personality Questionnaire—Brief Form (MPQ-BF; Patrick et al. 2002). The pre-screening protocol also included the *Social Closeness* scale to disguise the study’s focus on stress reactivity. The median *T* score on *Stress Reaction* was 40 with seven participants who scored at or below the median (*T* = 34–40) and six above the median (*T* = 48–66). In other words, there was a mix of participants ranging from 1.5 *SD* below the norm to 1.5 *SD* above the norm on the trait of *Stress Reaction*.

General exclusion criteria were smoking; upper respiratory infection, allergies or reflux disease affecting voice; hearing loss; pulmonary, cardiac, neurological, or endocrine diseases; current psychiatric or psychological treatment; and body mass index in the obese range (BMI > 30, not fitting into the scanner). Voice-specific exclusion criteria were a current or lifetime history of a voice disorder, vocal pathology, laryngeal trauma or neck surgery, previous voice therapy, professional singing or voice training. Vocal health status was determined by laryngeal videostroboscopy and clinically standard auditory-perceptual evaluation of voice production. Participants who did not achieve mid-membranous vocal fold closure during modal pitch and comfortable loudness were excluded. Two of the authors are certified speech-language pathologists (MD, JCS) and reviewed the exams independently. Complete agreement was required on a participant’s vocal health status before inclusion. The study was approved by the University of Kentucky Institutional Review Board as a deception study. All participants provided informed consent and were compensated monetarily for their time. Participants were fully debriefed about the true focus and nature of the research before exiting the study.

### Functional MRI paradigm

An event-related sparse-sampling design was used to study voice and speech production, capitalizing on the delayed task-related hemodynamic response in the brain (Perrachione and Ghosh 2013). The TR was seven seconds split into three seconds for volume acquisition and a silent delay of four seconds for speech production during which the scanner gradients were turned off (jittered 3.5–4.5 s). Meanwhile, the duration of volume acquisition was also used for the projection of task instructions onto a translucent screen (Silent Vision SV-6011 LCD, Avotec Inc., Stuart, FL) using E-Prime software (Psychology Software Tools Inc., Pittsburgh, PA). All participants wore MRI-compatible headphones with a microphone to communicate with the experimenters (Resonance Technology Inc., Northridge, CA).

The tasks included 30 trials of voiced sentence reading (Voice), 30 trials of whispered sentence reading (Whisper), and 40 trials of rest (fixation cross) distributed across two runs per experimental condition (No Stress vs. Stress). The trials for the voiced versus whispered task conditions were presented in a pseudo-randomized order within each run with the sentence stimuli repeated across trials. The stimuli were selected short sentences from the Consensus Auditory Perceptual Evaluation of Voice (Kempster et al. 2009) that were biased with voiced sounds (obligatory vocal fold vibration required for production): *The blue spot is on the key again* (production of every vowel sound in English), *We were away a year ago* (all voiced), *We eat eggs every Easter* (vowel onsets). Phonemes and syllables have been used in past fMRI research aimed at localizing the LMC (Brown et al. 2008; Simonyan et al. 2009). Here we chose sentences to capture more complex and naturalistic speech beyond simple laryngeal tasks (e.g. /ihi/). Further, the task contrast of Voice versus Whisper was designed to isolate phonation from articulatory aspects of speech production. The Voice task condition completely engaged the larynx, requiring the participant to use the full range of neuromuscular control to achieve medial compression and stiffness regulation of the vocal folds. On the other hand, the Whisper task condition required minimal and incidental movement of the vocal folds, relying instead on airflow turbulence through a narrowed glottis to generate acoustical sources of energy (Konnai et al. 2017). The same sentences were produced in both experimental conditions (No Stress vs. Stress) so that articulatory activity and language content were equivalent across task conditions. The expected net result would be the task-related cortical activity underlying laryngeal control for speech.

### Experimental stress induction

After our baseline conditions, social-evaluative stress was imposed using a modified public speaking stressor script extracted from the Trier Social Stress Test (Kirschbaum et al. 1993). Participants were told that they had to give a five-minute impromptu “speech” about why they are the best candidate for a position in a law firm. The script was modified such that it did not include a preparation phase and ended with the caveat that for control purposes there was a small chance that the prepared speech would not be delivered. Participants were then prompted to read the stimuli again while mentally preparing for their speeches. The scanner runs started and ended with visual cues indicating that the speech may start at any moment. Elements of time pressure, uncertainty, and social evaluation (participants were told that three experimenters would be observing their performances) were used to uphold the psychological tension during the stressor task period. None of the participants actually delivered a speech.

### Measurements

#### Endocrine measures

**Fig. 1:**
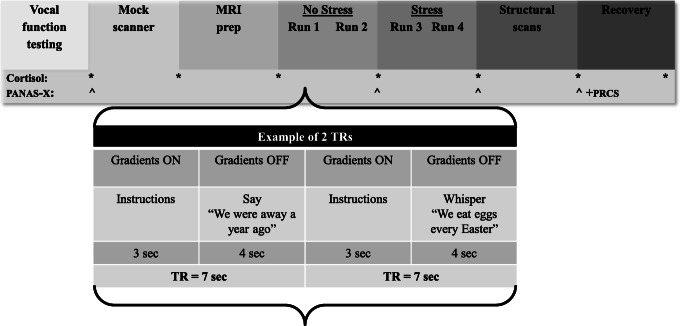
Timeline of procedures along with outcome measures and an example of the event-related sparse-sampling design. PANAS-X = Positive and Negative Affect Scales–Expanded Form; PRCS = Personal Report of Confidence as a Speaker

A key limitation of many stress studies is a failure to validate the stress response with cortisol measures (Dedovic et al. 2009a), an objective marker of the biological stress response (Dickerson and Kemeny 2004). Seven samples were collected with Salimetrics Oral Swabs (Salimetrics, State College, PA) in approximately10-minute intervals starting 45–60 min before the onset of the stressor (first sample immediately before mock scanner training) until 40–50 min post stressor (Fig. 1). Cortisol peaks 20–40 min post-stressor and all cortisol samples were collected in the late afternoon/early evening (4:00–7:00 p.m.) to best capture a consistent cortisol response (Dickerson and Kemeny 2004). When participants were in the MRI scanner, saliva oral swab tubes were placed in the participant’s hand between runs. The routine of placing the oral swab in their mouth and back in the tube was practiced in the mock scanner before actual data collection.

#### Psychometric measures of personality

The MPQ-BF was chosen for the pre-screening because it contains a separate scale to measure *Stress Reaction* (dimension *Negative Emotionality*). For a full personality assessment, participants completed the NEO-Personality Inventory—Revised (NEO-PI-R; Costa and McCrae 1992) during screening. The 240-item NEO-PI-R reflects the integrative trait taxonomy with the factors of *Neuroticism*, *Extraversion*, *Openness*, *Agreeableness*, and *Conscientiousness* with each factor comprising several subscales. Each question was answered on a 5-point Likert scale ranging from *strongly disagree* to *strongly agree*. Participants also completed the BIS/BAS personality scales (Carver and White 1994) and the Rosenberg self-esteem scale (Rosenberg 1989) during screening. The 24-item BIS/BAS scales (7 items BIS) capture the behavioral motivational systems behavioral inhibition and behavioral activation. Questions were answered on a 4-point Likert scale ranging from *very true for me* to *very false for me*. Questions on the 10-item Rosenberg scale were answered on a 4-point Likert scale ranging from *strongly agree* to *strongly disagree*. A low Rosenberg score has been shown to be linked to greater cortisol responsiveness during social-evaluative stressor exposure (Dickerson and Kemeny 2004).

#### Psychoemotional state measures

Throughout the experiment participants provided ratings of their emotional state using selected items from the Positive and Negative Affect Scale–Expanded Form (PANAS-X; Watson and Clark 1994). Participants rated the six items of the basic negative emotion scale for fear on a 5-point Likert scale ranging from *very slightly or not at all* to *extremely* after training in the mock scanner, via intercom after the baseline phase and stress phase, and in writing for the recovery phase. After participants left the scanner, they also completed the 30-item *true/false* Personal Report of Confidence as a Speaker (PRCS; Paul 1966) to capture the degree of fear of public speaking they experienced during stressor exposure.

#### Vocal function measures

During the screening, participants completed the 30-item Voice Handicap Index (Jacobson et al. 1997) using a 5-point Likert scale from *never* to *always*, which assessed the impact that a voice disorder may have on perceived quality of life in functional, physical, and emotional domains. Immediately before the MRI session, data on aerodynamic vocal function were collected using the automated computer-based Phonatory Aerodynamic System (PAS, model 6600, KayPentax, Montvale, NJ). The voicing efficiency protocol, consisting of a string of five plosive consonant-vowel syllables (/pa/), was used to determine mean peak subglottal pressures (cm H_2_O), mean airflow during voicing (L/s), and laryngeal airway resistance (R_law_ = cm H_2_O/[L/s]). R_law_ is considered a proxy measure for vocal effort and laryngeal muscle activity. The implications of an elevated R_law_ would be an increased risk for excessive vocal effort and fatigue, a commonplace outcome in occupational voice users (Roy et al. 2004).

### Procedures

Figure 1 shows the timeline of the experimental procedures. Prior to the experiment, participants were asked to abstain from alcohol (12 h), exercise (on the day of the experiment), a large meal (2 h), and caffeine (3 h) (MacArthur Research Network on Socioeconomic Status and Health 2000; Shapiro et al. 1996). Data on voicing efficiency were collected first. Participants were then escorted to the University of Kentucky’s MRI & Spectroscopy Center’s mock scanner where they were trained on the tasks and familiarized with the scanning environment. After training, participants were set up in the research MRI scanner. During the No Stress condition (first two runs), participants read short sentences in voiced and whispered manners or rested quietly (conditions Voice No Stress [VoiceNS], Whisper No Stress [WhisperNS]). After that, participants were told for the first time that they had to give a 5-min impromptu speech while being observed and evaluated by a panel of three external individuals. During the Stress condition that followed (third and fourth runs), participants were prompted to again read sentences in voiced and whispered manners, however this time, while waiting for their prompt to start their speech (conditions Voice Stress [VoiceS], Whisper Stress [WhisperS]). After the Stress runs, the “speech” delivery was waived and participants were fully debriefed as to the actual purpose of the research. Participants provided ratings of their negative emotional state using the PANAS-X after training in the mock scanner, after the No Stress condition, after the Stress condition in the scanner, and lastly after the recovery period outside the scanner followed by completion of the PRCS. Seven salivary cortisol samples were collected with oral swabs in approximately 10-min intervals throughout the experiment.

### FMRI data acquisition

The fMRI data were acquired on a Siemens Magnetom Trio Tim 3T scanner (Siemens, Erlangen, Germany) equipped with a 32-channel head coil. Data from four functional runs were collected using T-2*-weighted gradient echo echo-planar imaging (EPI) scans (acquisition parameters: TR = 7.0 s, TA = 3.0 s, delay = 4.0 s, TE = 30 ms, flip angle = 81°, FoV = 224 mm × 224 mm, 64 × 64 matrix, slice thickness = 3.5 mm, voxel resolution = 3.5 mm × 3.5 mm × 3.5 mm, bandwidth = 2056 Hz/Px, 40 interleaved axial slices providing whole-brain coverage, number of volumes = 224). A whole-brain high-resolution anatomical volume was acquired using a T-1weighted magnetization-prepared rapid gradient echo (MPRAGE) sequence (acquisition parameters: TR = 2100 ms, TE = 2.93 ms, TI = 1100 ms, flip angle = 12°, FoV = 224 mm × 256 mm, voxel resolution = 1 mm isotropic voxels). At the beginning of each run, three TRs with no data were discarded to allow for stabilization of longitudinal magnetization.

### Data analysis

#### Data analysis of endocrine measures

Salivary cortisol samples were stored in a -80^o^ C freezer and then analyzed in duplicate at the University of Kentucky Clinical Research Development and Operations Center Core Biochemical Analysis Laboratory (Salivary Cortisol Kit 1–3002, Salimetrics, State College, PA). A participant was a stress responder when the cortisol area under the curve with reference to increase (AUC_I_) (Pruessner et al. 2003) was positive. Participants were categorized as non-responders when AUC_I_ levels were zero or negative. Personality characteristics and aerodynamic vocal function were correlated with these salivary responses. In addition, non-responders and stress responders were descriptively compared on personality characteristics and aerodynamic vocal function.

#### Data analysis of psychometric, psychoemotional, and vocal function measures

For the psychometric measures, descriptive statistics were determined for the two MPQ-BF subscales *Stress Reaction* and *Social Closeness*, the five factors of the NEO-PI-R, the BIS subscale, and the Rosenberg scale. The scores on fear ratings (PANAS-X) for the total group were statistically compared across conditions (No Stress, Stress, recovery) using a within-subjects ANOVA. The *p* value was set at .05. The statistical analyses were performed with SPSS Statistics for Windows, Version 21.0 (IBM Corp., Armonk, NY). Descriptive statistics were determined for fear of public speaking (PRCS). Voicing efficiency data were analyzed by averaging the middle three productions of /pa/ from a set of five to determine values for subglottal pressure, airflow, and R_law_ following recommendations by Solomon and Helou (2013).

#### FMRI data analysis

Preprocessing, analysis, and presentation of fMRI data were completed with Analysis of Functional NeuroImages software, version AFNI_2011_12_21_1014 (http://afni.nimh.nih.gov/afni) (Cox 1996) with the exception of the preparation of field maps and the subsequent correction of B0 distortions in the EPI data, which was carried out using FSL version 5.0.8 (FMRIB’s Software Library, www.fmrib.ox.ac.uk/fsl) (Jenkinson et al. 2012).

##### Preprocessing

Data were converted from DICOM format to FSL NIfTI format using *MRIConvert* (http://lcni.uoregon.edu/downloads/mriconvert/mriconvert-and-mcverter) and also from DICOM to AFNI format using *to3d*. Field maps were prepared using the FSL tool *fsl_prepare_fieldmap*. The FSL command line tool *fugue* was then used to unwarp B0 distortions in the EPI data (EPI files were converted in AFNI to apply the correct volume acquisition timing for sparse sampling, then converted to NIfTI format using *3dAFNtoNIFTI*). The processing pipeline was generated using *uber_subject.py* (version 0.39) that ran the *afni_proc.py* super-script (Cox 2012). In a single transformation, the EPI and high-resolution anatomical images were co-registered, normalized to MNI template space (MNI Avg152, T1 1x1x1 mm) using non-linear warping, and the data were motion corrected using cubic polynomial interpolation. The reference volume for volume registration (realignment) was the last volume of the last run because the anatomical dataset was acquired after the EPI. We applied a 4.0 mm FWHM Gaussian filter to smooth the data (on top of the existing blur) and obtained estimates of the final blur of the data (approximately 6 mm). A moderate smoothing value was chosen to balance the signal-to-noise ratio. The final blur estimates were averaged across participants and used in *3dClustSim* to estimate the probability of false-positive clusters. Finally, a brain mask was created from the EPI data (dilate 1 voxel) and each voxel was scaled to a mean of 100 for units in percent signal change. Slice timing correction was not applied to the EPI data because a sparse-sampling design was used for volume acquisition and thus, data acquisition was discontinuous during the TR.

##### Model design and estimation

Regression analysis was completed using a fixed shaped regression using AFNI BLOCK, which convolves an incomplete gamma function with a boxcar function, where each has a height of 1. The BLOCK curve lasts about 15.8 s longer than the stimulus duration (3.5 s). Each beta weight represents the peak magnitude of the response to the entire stimulus block. First, we examined the main effects of each condition No Stress (NS) and Stress (S) condition: VoiceNS, VoiceS, WhisperNS, WhisperS. Then, the program *uber_ttest.py* (version 1.1) was used at the group level to test the following contrasts: VoiceS-VoiceNS, WhisperS-WhisperNS, WhisperS-VoiceS, and WhisperNS-VoiceNS. Clusters were corrected for multiple comparisons to achieve a family-wise error (FWE) rate of *p* < .05.

#### ROI data analysis

Anatomical regions of interest (ROI) were defined a priori and primarily informed by evidence about primary and secondary vocal areas (Brown et al. 2009) as well as the functional connectome of speech control under consideration of laterality (Fuertinger et al. 2015; Simonyan and Horwitz 2011) and corticolimbic and limbic regions involved in stress responses (Dedovic et al. 2009b; Lorberbaum et al. 2004; Wager et al. 2009). Masks were created using the AFNI adaptions of the Eickhoff-Zilles maximum probability maps (areas 4p and 6) and macro label maps (postcentral gyrus, SMA, MFG, ACC, MCC, insula, thalamus, putamen, amygdala, HC, and lobule VI of the cerebellum) (Eickhoff et al. 2005). These maps were also used to label anatomical regions for the whole brain analyses. In addition, a bilateral ROI mask for the PAG with the stereotaxic Talairach space coordinates x-axis − 8 to +8, y-axis − 26 to −33, and z-axis − 4 to −16 was drawn manually using AFNI.

BOLD percent signal changes were extracted for each ROI for the Voice No Stress and Voice Stress conditions and were correlated with cortisol data using Spearman’s rho to gain insight about individual differences in BOLD reactivity as a function of perceived stress. In a second step, BOLD data that correlated significantly with AUC_I_ values were correlated with personality characteristics and aerodynamic vocal function. Because of the exploratory nature of investigating individual differences, the *p* value was < .1 (two-tailed).

## Results

### Salivary cortisol

**Fig. 2:**
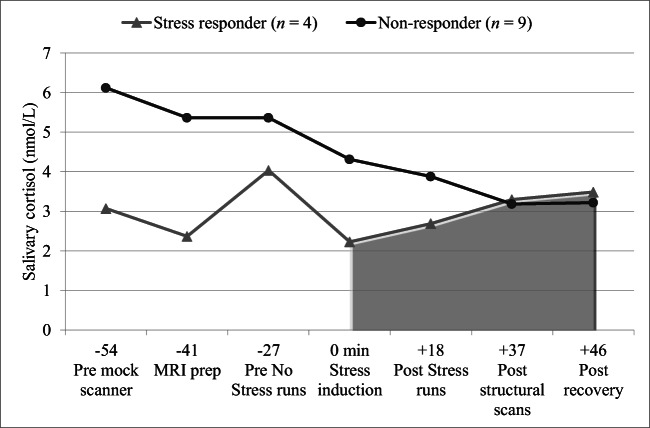
Time course of salivary cortisol levels in stress responders and non-responders pre and post stress induction. Time zero indicates the cortisol sample immediately before experimental stress induction. Cortisol peaks 20–40 min post stressor. Practice in the mock scanner elicited reactivity in some participants. Therefore, area under the curve with respect to increase (AUC_I_) was calculated using the last four samples

Calculations of AUC_I_ for salivary cortisol were based on samples collected immediately before the onset of the stressor until approximately 50 min after the stressor event. The first three cortisol samples were not included in our analysis because of possible contamination by the participants’ experience in the mock scanner from which they would have recovered before stressor induction. Four participants showed positive AUC_I_ values (stress responders) while nine showed negative AUC_I_ values (non-responders). Figure 2 shows cortisol data for the stress responders and non-responders over time.

### Psychometric measures

Table 1 shows the personality characteristics of the total sample (including significant correlations with AUC_I_) as well as those of stress responders and non-responders. Stress responders scored higher than non-responders on all scales of Neuroticism and Openness (except Ideas and Values), lower on all scales of Extraversion (except for Excitement-Seeking) and Conscientiousness, and mixed on Agreeableness (lower on Trust, Compliance, Tender-Mindedness and higher on Straightforwardedness, Altruism, Modesty). Further, stress responders had lower mean scores on behavioral inhibition and self-esteem. Significant correlations with AUC_I_ values in the total sample were found for Extraversion-Activity (−.58, *p* = .037), Agreeableness-Modesty (.51, *p* = .077), and Conscientiousness-Achievement Striving (−.65, *p* = .016).

**Table 1:** The distribution of scores on personality tests in the total sample (*n* = 13) and stress responders (*n* = 4) and non-responders (*n* = 9). Significant correlations with salivary cortisol (AUC with respect to increase) in response to stressor exposure are starred

	Total sample	Cortisol subgroups	
		Stress responder	Non-responder
Measurement tool	*M (SD)*	*M (SD)*	*M (SD)*
MPQ-BF^a^			
Stress Reaction	46.0 (10.8)	51.3 (10.8)	43.7 (10.6)
Social Closeness	54.2 (11.5)	49.3 (8.4)	56.3 (12.4)
NEO-PI-R^a^			
Neuroticism	52.9 (12.9)	63.3 (5.7)	48.3 (12.6)
Anxiety	55.3 (10.7)	66.3 (7.2)	50.4 (8.0)
Angry Hostility	52.2 (11.7)	60.8 (6.3)	48.4 (11.7)
Depression	49.8 (11.8)	59.5 (6.6)	45.6 (11.2)
Self-Consciousness	52.9 (12.6)	60.5 (6.0)	49.6 (13.5)
Impulsiveness	51.9 (12.9)	55.3 (9.2)	50.4 (14.5)
Vulnerability	51.4 (11.1)	55.8 (11.4)	49.4 (11.1)
Extraversion	56.7 (14.9)	52.8 (4.0)	58.4 (17.7)
Warmth	50.6 (16.1)	47.0 (8.0)	52.2 (18.9)
Gregariousness	53.4 (15.7)	47.3 (9.9)	56.1 (17.5)
Assertiveness	54.2 (12.8)	46.8 (11.8)	57.6 (12.3)
Activity*	54.1 (10.1)	48.3 (9.4)	56.7 (9.8)
Excitement-Seeking	57.8 (10.2)	63.5 (6.6)	55.2 (10.8)
Positive Emotions	56.0 (11.4)	54.8 (11.1)	56.6 (12.1)
Openness	58.1 (9.8)	62.8 (10.5)	56.0 (9.3)
Fantasy	60.3 (11.6)	68.0 (10.5)	56.9 (10.9)
Aesthetics	54.5 (13.2)	59.0 (11.8)	52.6 (14.0)
Feelings	56.7 (10.1)	58.8 (9.4)	55.8 (10.8)
Actions	49.9 (10.6)	53.5 (12.4)	48.3 (10.1)
Ideas	55.7 (7.9)	55.5 (7.9)	55.8 (8.4)
Values	54.1 (10.1)	53.5 (12.9)	54.3 (9.5)
Agreeableness	47.0 (9.4)	46.8 (3.9)	47.1 (11.2)
Trust	47.2 (12.2)	38.8 (8.3)	50.9 (12.1)
Straightforwardedness	47.6 (8.4)	48.8 (6.2)	47.1 (9.5)
Altruism	51.4 (11.6)	54.5 (8.7)	50.0 (12.9)
Compliance	45.9 (8.9)	42.5 (6.0)	47.4 (9.9)
Modesty	49.4 (11.4)	58.0 (15.5)	45.6 (7.1)
Tender-Mindedness	47.8 (7.5)	47.0 (2.4)	48.2 (9.1)
Conscientiousness	46.6 (17.0)	33.8 (9.8)	52.3 (16.7)
Competence	48.6 (14.0)	42.0 (7.3)	51.6 (15.6)
Order	46.7 (12.8)	38.0 (11.7)	50.6 (11.9)
Dutifulness	48.0 (12.7)	41.5 (5.0)	50.9 (14.2)
Achievement Striving*	49.5 (15.8)	37.5 (11.8)	54.8 (14.8)
Self-Discipline	43.4 (16.1)	28.3 (7.9)	50.1 (14.1)
Deliberation	49.2 (15.0)	42.8 (11.6)	52.1 (16.0)
BIS/BAS			
BIS subscore^b^	20.5 (3.2)	18.8 (4.6)	21.2 (2.2)
Rosenberg^c^			
Total score	22.2 (6.0)	17.8 (6.3)	24.1 (4.9)

^a^MPQ-BF and NEO-PI-R scores are *T* scores.

^b^BIS ranges from 7 to 28. ^c^Rosenberg scores range from 0 to 30 with < 15 indicating low self-esteem

*Negative correlations with salivary cortisol AUC with respect to increase; significant at the .05 level

### Psychoemotional stress reactivity

**Fig. 3:**
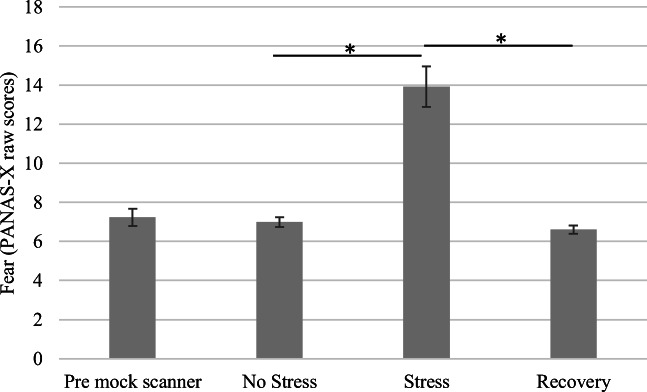
Ratings of perceived fear based on the Positive and Negative Affect Scales–Expanded Form (PANAS-X) by condition. Stress ratings were significantly greater than ratings for either No Stress or recovery, *p* < .001

The scores for the fear subscale (PANAS-X) in the total sample were compared among the No Stress (*M* = 7.0, *SD* = 0.9), Stress (*M* = 13.9, *SD* = 3.7), and recovery conditions (*M* = 6.6, *SD* = 0.8). The main effect of condition on ratings of fear was significant: *F*(2, 24) = 46.46, *p* < .001, partial η^2^ = 0.80. Planned comparisons revealed that ratings for the Stress condition were significantly greater than ratings for either the No Stress or recovery conditions, *p* < .001. The ratings for the baseline and recovery phases were not significantly different. Figure 3 shows the results. The mean Personal Report of Confidence as a Speaker (PRCS) score in the total sample was 15.7 (*SD* = 6.5). Stress responders had a mean of 20.0 (*SD* = 1.4) and non-responders a mean of 13.8 (*SD* = 7.1) (possible maximum score of 30 indicating greater fear of public speaking).

### Vocal function

The mean Voice Handicap Index scores were 17.0 (*SD* = 16.4) in the total sample, 26.8 (*SD* = 22.2) for stress responders, and 12.7 (*SD* = 12.3) for non-responders. Regarding voicing efficiency analyses, data from one participant were missing due to experimenter error and subglottal pressure data from four participants did not meet quality criteria. For the remaining twelve participants, airflow during voicing was 0.14 L/s (*SD* = 0.05). Subglottal pressure (P_sub_) and laryngeal airway resistance (R_law_) for eight participants with complete datasets were 7.64 cm H_2_O (*SD* = 1.26) and 71.31 cm H_2_O/(L/s) (*SD* = 49.17), respectively. Thus, voicing efficiency data were within the normal range (Zraick et al. 2012). Stress responders had lower airflow during voicing than non-responders: stress responders P_sub_ 7.10 cm H_2_O (*SD* = 1.74) (*n* = 3), airflow during voicing 0.10 L/s (*SD* = 0.04), and R_law_ 91.45 cm H_2_O/(L/s) (*SD* = 79.87) (*n* = 3); non-responders P_sub_ 7.97 cm H_2_O (*SD* = 0.95) (*n* = 5), airflow during voicing 0.17 L/s (*SD* = 0.05), and R_law_ 59.23 cm H_2_O/(L/s) (*SD* = 23.56) (*n* = 5). Further, intensity and *f*_o_ differed between stress responders [SPL during voicing 76.4 dB (*SD* = 4.1), mean *f*_o_ 213.1 Hz (*SD* = 40.23)] and non-responders [SPL during voicing 78.1 dB (*SD* = 3.6), mean *f*_o_ 190.8 Hz (*SD* = 25.4)]. Correlations with cortisol reactivity (AUC_I_) values were non-significant.

### Neuroimaging data

#### Whole brain analyses

#### Brain activation in the total sample

**Fig. 4:**
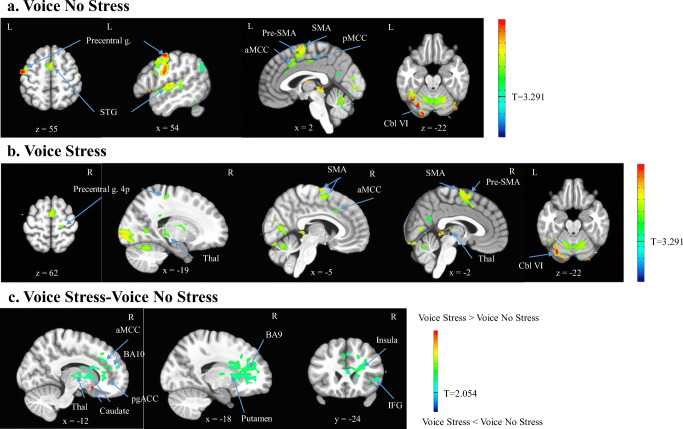
BOLD activation maps for the conditions (**a**). Voice No Stress (VoiceNS) minus rest, (**b**). Voice Stress (VoiceS) minus rest, and the contrast (**c**). VoiceS minus VoiceNS over rest. The *t* statistical parametric maps were thresholded at *p* = .001 (VoiceNS and VoiceS) and *p* = .04 (contrast VoiceNS vs. VoiceS) to achieve a family-wise error corrected *p* < .05. The contrast showed stressor-induced activations (red) and deactivations (green) in the caudate and deactivations (green/blue) in the right perigenual anterior cingulate cortex (pgACC), anterior middle cingulate cortex (aMCC), inferior frontal gyrus (IFG), BA 9, BA 10, insula, putamen, and thalamus (Thal). Precentral g. = precentral gyrus, STG = superior temporal gyrus, SMA = supplementary motor area, Cbl VI = cerebellum lobule VI

Figure 4 shows the brain areas activated in the total sample for the VoiceNS and VoiceS conditions separately, as well as the contrast of the two conditions after removal of baseline activation (rest). The contrast showed stressor-induced activations and deactivations in the caudate and deactivations in the right perigenual ACC (pgACC), anterior MCC (aMCC), inferior frontal gyrus (IFG, pars triangularis), BA 9, BA 10, insula, putamen, and thalamus. Tables 2 and 3 present an exhaustive list of all activated regions.

**Table 2:** Activated brain regions for the Voice No Stress minus rest and Voice Stress minus rest conditions in the total sample (*n* = 13, *t* = 3.291, uncorrected *p* = .001, family-wise error corrected *p* < .05, minimum cluster size 7 voxels)

Anatomical Region	Peak MNI Coordinates			Cluster Size in Voxels	*p* value, corrected
	x	y	z		
Voice No Stress					
L Precentral g.	−54	−9	55	1199	< .001
L Cerebellum (VI)	−33	−72	−22	865	< .001
R Superior temporal g.	68	−16	13	423	< .001
L Inferior parietal lobule	−51	−72	44	82	< .001
L Superior parietal lobule	−26	−69	48	18	< .01
R Brainstem	5	−30	−43	15	< .01
L Precuneus	−2	−58	23	14	< .01
L Fusiform g.	−26	−37	−15	11	< .01
L Caudate n.	−16	19	20	11	< .01
L Middle occipital g.	−26	−72	30	9	< .02
R Cuneus	5	−100	27	9	< .02
L Middle cingulate g.	−2	−16	44	8	< .03
R Lingual g.	30	−48	−8	7	< .05
L Cuneus	−16	−62	20	7	< .05
L Medial frontal g.	−23	40	16	7	< .05
Voice Stress					
L STG	−68	−9	6	657	< .001
R STG	68	−16	13	519	< .001
L Cerebellum (VI)	−33	−72	−22	290	< .001
R Inf. occipital g.	26	−90	−22	273	< .001
R SMA	2	−6	72	97	< .001
L Angular g.	−54	−69	37	28	< .001
L Middle occipital g.	−30	−72	27	18	< .01
L Inf. temporal g.	−47	−51	−26	17	< .01
L Angular g.	−40	−62	30	14	< .01
Cerebellar vermis	2	−41	2	11	< .01
L Precuneus	−2	−62	27	11	< .01
L Inf. parietal lobule	−30	−51	44	11	< .01
R Precentral g. (4p)	19	−27	62	10	< .02
R MCC	5	19	34	9	< .02
R Cerebellum (VIIa)	47	−72	−29	7	< .05
R Lingual g.	2	−93	−15	7	< .05
L ParaHippocampal g.	−26	−44	−8	7	< .05
R Caudate n.	26	−41	20	7	< .05

*MCC* middle cingulate cortex, *MTG* middle temporal gyrus, *SMA* supplementary motor area, *STG* superior temporal gyrus

**Table 3:** Activated brain regions for the contrasts Voice Stress minus Voice No Stress over rest and Whisper Stress minus Whisper No Stress over rest in the total sample (*n* = 13, *t* = 2.054, uncorrected *p* = .04, family-wise error corrected *p* < .05, minimum cluster size 469 voxels and 623 voxels, respectively)

Anatomical region	Peak MNI Coordinates			Cluster Size in Voxels	*p* value, corrected
	x	y	z		
Voice Stress > Voice No Stress					
R Caudate n.	12	15	−12	472	< .05
Whisper Stress < Whisper No Stress					
L ParaHippocampal g.	−2	−34	2	727	< .05

**Fig. 5:**
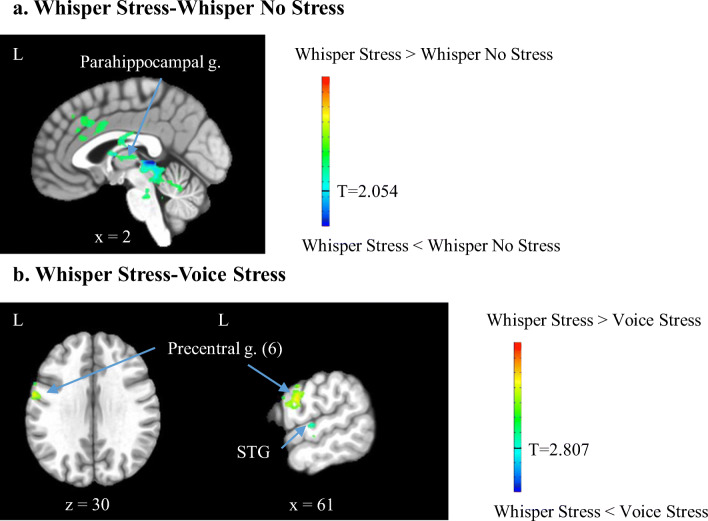
BOLD activation maps for the contrasts (**a**). Whisper Stress (WhisperS) minus Whisper No Stress (WhisperNS) over rest and (**b**). WhisperS minus VoiceS over rest. The *t* statistical parametric maps were thresholded at *p* = .04 (WhisperS vs. WhisperNS) and *p* = .005 (WhisperS vs. VoiceS) to achieve a family-wise error corrected *p* < .05. For the Whisper condition, stressor exposure resulted in deactivation of the parahippocampal gyrus (parahippocampal g.). Contrasting Whisper and Voice during Stress showed increased activations in the precentral gyrus (precentral g. area 6) and decreased activations in the superior temporal gyrus (STG)

Figure 5 shows BOLD activation maps for the contrasts of WhisperS minus WhisperNS over rest as well as WhisperS minus VoiceS over rest. For the Whisper conditions, stressor exposure resulted in deactivation of the parahippocampal gyrus (Table 3). Contrasting Whisper and Voice conditions during Stress, with the aim of revealing laryngeal-related activations, showed greater activations in the precentral gyrus (area 6) and less activations in the STG for the Whisper than the Voice condition during Stress (Table 4). Activity in area 6 was in close proximity to the dorsolateral LMC described by Brown et al. (2008, 2009). In addition, Table 4 lists brain regions activated for the contrast of Whisper and Voice during the No Stress condition. For this contrast, brain activations were greater in the left MFG and right precentral gyrus for the Whisper than the Voice condition.

**Table 4:** Activated brain regions for the contrasts Whisper No Stress minus Voice No Stress over rest and Whisper Stress minus Voice Stress over rest in the total sample (*n* = 13, *t* = 2.807, uncorrected *p* = .005, family-wise error corrected *p* < .05, minimum cluster size 17 voxels and 20 voxels, respectively)

Anatomical region	Peak MNI Coordinates			Cluster Size in Voxels	*p* value, corrected
	x	y	z		
Whisper No Stress > Voice No Stress					
L Middle frontal g.	−61	1	41	99	< .001
R Precentral g.	68	−16	41	77	< .001
Whisper Stress < Voice Stress					
L Superior temporal g.	−68	−13	2	35	< .02
Whisper Stress > Voice Stress					
L Precentral g. (6)	−61	−2	30	28	< .03

#### ROI analyses

**Fig. 6:**
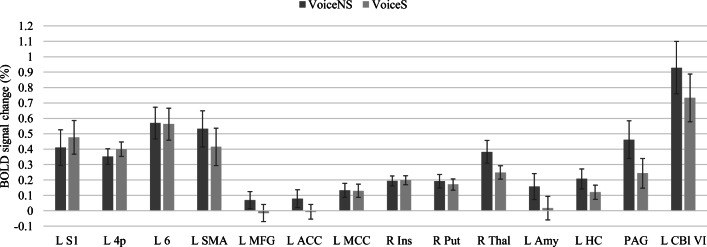
Brain responses in mean percent BOLD signal change in regions of interest (ROI) for voice productions during the No Stress and Stress conditions. The ROIs represent regions involved in voice and speech production and stress responses with consideration of lateralization. L/R = left/right, S1 = postcentral gyrus, 4p = posterior part of area 4, 6 = area 6, SMA = supplementary motor area, MFG = middle frontal gyrus, ACC = anterior cingulate cortex, MCC = middle cingulate cortex, Ins = insula, Put = putamen, Thal = thalamus, Amy = amygdala, HC = hippocampus, PAG = periaqueductal gray, and Cbl (VI) = Lobule VI of the cerebellum

Figure 6 shows mean percent BOLD signal changes in ROIs for the VoiceNS and VoiceS conditions. BOLD signals decreased in the corticolimbic areas MFG, ACC, amygdala, HC, thalamus, and PAG. In the primary vocalization areas, L area 4p motor activity increased and L SMA and L cerebellum lobule VI activity decreased. In the secondary vocalization areas, responses in the MCC and insula remained stable overall but activity in the putamen and thalamus decreased. In summary, stressor exposure was characterized by increases in sensorimotor activity and decreases in SMA, cerebellum, and limbic activity.

##### Correlations of BOLD activations in ROIs with salivary cortisol, personality, and aerodynamic vocal function

**Fig. 7:**
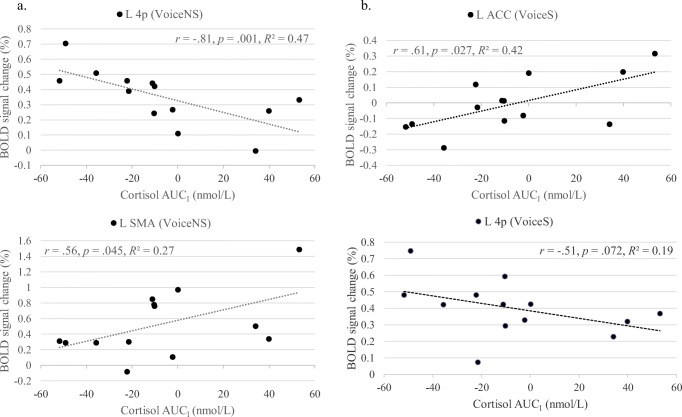
Correlations between the cortisol response to the stressor (area under the curve with respect to increase [AUC_I_ in nmol/L]) and mean percent BOLD signal changes during the conditions (**a**). Voice No Stress (VoiceNS) and (**b**). Voice Stress (VoiceS) in the left (L) area 4p, L supplementary motor area (SMA), and L anterior cingulate cortex (ACC)

Figure 7 shows all significant correlations at *p* < .05 and L area 4p correlations between salivary cortisol responses (AUC_I_) and mean percent BOLD signal change in ROIs during the VoiceNS and VoiceS conditions. For VoiceNS, there was a strong negative correlation for cortisol reactivity with signal changes in the L area 4p (*r* = −.81, *p* = .001) and moderate positive correlations with the L SMA (*r* = .56, *p* = .045). For VoiceS, a moderately strong correlation emerged for the L ACC (*r* = .61, *p* = .027). Further, multiple moderate correlations were significant at *p* < .1 including, in order of significance, right thalamus (*r* = .54, *p* = .058), L 4p (*r* = −.51, *p* = .072), L MCC (*r* = .50, *p* = .082), and L HC (*r* = .50, *p* = .086).

The above mentioned ROIs were significantly correlated with selected personality traits and aerodynamic measures. For the active control condition, Voice No Stress, L area 4p was strongly correlated with Agreeableness-Modesty (*r* = −.71, *p* = .006) and moderately correlated with airflow (*r* = .68, *p* = .015), Neuroticism-Depression (*r* = −.65, *p* = .016), self-esteem (*r* = .63, *p* = .020), Extraversion-Activity (*r* = .60, *p* = .029), Conscientiousness-Achievement Striving (*r* = .57, *p* = .042), and Extraversion (*r* = .50, *p* = .079). In addition, airflow was also moderately correlated with PAG activity (*r* = −.59, *p* = .046) but with no further brain region. Further, L SMA was also moderately correlated with Extraversion-Activity (*r* = −.51, *p* = .073) as well as Agreeableness-Altruism (*r* = .51, *p* = .072) and Agreeableness-Straightforwardedness (*r* = .48, *p* = .100).

For the active task condition, Voice Stress, L area 4p was moderately correlated with BIS scores (*r* = .67, *p* = .013) and again Conscientiousness-Achievement Striving (*r* = .59, *p* = .035). Further, the L ACC was moderately correlated with P_sub_ (*r* = −.67, *p* = .071), the R thalamus was moderately correlated with Extraversion-Gregariousness (*r* = .49, *p* = .088), and the L hippocampus was moderately correlated with Openness-Ideas (*r* = .69, *p* = .009), R_law_ (*r* = −.67, *p* = .071), Agreeableness-Straightforwardedness (*r* = .64, *p* = .018), Agreeableness (*r* = .53, *p* = .060), and Agreeableness-Compliance (*r* = .49, *p* = .092). The L MCC did not have significant correlations.

To summarize, the lower a participant’s L area 4p activity was, the lower the person’s Extraversion-Activity, Conscientiousness-Achievement Striving, self-esteem, and behavioral inhibition scores and the higher the person’s Neuroticism-Depression and Agreeableness-Modesty scores. Lower Extraversion-Activity also correlated with greater AUC_I_ values and L SMA activations. With regard to aerodynamic measures, lower L area 4p activity correlated with lower airflow during No Stress. During Stress, lower P_sub_ and greater R_law_ correlated with greater L ACC and lower L HC activity, respectively.

## Discussion

### Effects of stress on brain activations in the total sample

The first aim of this study was to determine the effect of social-evaluative stress on the central neural control of phonation underlying speech production. First, brain activations during the VoiceNS condition were largely consistent with the known speech production network underlying sentence reading (Fuertinger et al. 2015; Simonyan and Fuertinger 2015) apart from activations for visual processing, memory, and reading. The activated clusters encompassed the LMC (area 4p), IFG, SMA, STG, cingulate cortex, putamen, thalamus, and cerebellum (lobule VI). The stress induction was successful as shown by a significant increase in perceived fear during speech anticipation. The task contrast (VoiceS-VoiceNS) revealed that stressor exposure was characterized by a peak *activation* in the right caudate, as predicted, with concomitant *deactivations* in the bilateral pgACC and aMCC, and right IFG, BA 9, BA 10, insula, putamen, and thalamus. Thus, a significant effect on primary or premotor cortices was not detected.

The key impact of stressor exposure on the vocal control system occurred in areas considered secondary to phonation control, specifically *deactivations* in the ACC and MCC, insula, putamen, and thalamus. Except for the direction of the activation changes, our findings parallel those from an early PET study by Paus et al. (1993) whose experimental design required a simple one-word response, which was or was not in conflict with an overlearned stimulus-response alternative. Their study yielded primarily activations in the left paracingulate gyrus (BA 32/8) as well as in the rostral ACC (BA 24), BA 10, and BA 9/45. Our predictions were partially based on a study by Lorberbaum et al. (2004) that compared the effects of anticipation of public speaking in healthy controls and individuals with generalized social phobia. Our results are in striking concordance with those in the social phobia group instead of the control group. The social phobia group showed less activation in the left aMCC, medial PFC (BA 8,32), and dlPFC (BA 9) than the control group during stressor exposure. The authors concluded that limbic hyperactivation with cortical hypoactivation led to their participants not being able to “think clearly” (p. 2703). A reason for the activation directions of our results may be that we pre-selected our sample to include a comparable number of individuals who scored above and below the norm on stress reactivity instead of a random distribution.

The ACC and MCC are key regions because of potentially degrading effects on voice production. The ACC is involved in voice initiation and emotions underlying prosody (Simonyan and Horwitz 2011). However, simple tasks such as syllable productions do not generally evoke these activations whereas complex tasks such as reading or speaking do engage the prefrontal cortex and involve prosody and emotional vocalizations mediated by the cingulate cortex (Fuertinger et al. 2015; Simonyan and Fuertinger 2015; Simonyan and Horwitz 2011; Simonyan et al. 2009). The ACC and MCC transform intentions into actions via the neuromotor system, integrate cognitions via the lateral PFC, and modulate fear and arousal via the thalamus, amygdala, and PAG (Etkin et al. 2011; Paus 2001; Vogt 2016). The aMCC may influence vocal behavior through its role in conflict monitoring and (willed) action selection as the dorsal aMCC is pivotal to action anticipation, response initiation, and monitoring of ongoing action outcomes (Etkin et al. 2011; Vogt 2016).

Thus, the relative drop in activation in the aMCC may be linked to diminished guidance for motor choices and consequently disrupted implementation of voice as the aMCC (a) co-activates with the lateral PFC and MFG (Paus 2001; Paus et al. 1993), (b) connects with the SMA (Etkin et al.), and (c) maintains reciprocal connectivity with the LMC (Simonyan and Horwitz 2011). The LMC also has reciprocal connectivity with the dlPFC as well as with the insula and thalamus (Simonyan and Horwitz 2011). While the anterior insula appears to be involved in both articulation and phonation, its exact vocal motor role is still debated (Brown et al. 2009). The ventral tier nuclei of the thalamus has a well-established role in vocal sensorimotor coordination of learned voice production including the integration of information from the basal ganglia and cerebellum (Simonyan and Horwitz 2011). The putamen, which is also involved in learned voice production, often co-activates with the thalamus but only receives input from the LMC and does not feed back to the LMC (Simonyan and Horwitz 2011).

Most relevant in the context of stress, subcortical structures modulate the cognitive motor input received by the ACC and MCC in challenging situations, leading either to the facilitation or suppression of motor responses (Paus et al. 1993). The ACC and MCC directly connect with limbic structures including the amygdala, PAG, and HY (Etkin et al.; Vogt 2016). The pgACC, which is involved in emotion and autonomic regulation (Vogt 2016), can exert top-down control to inhibit negative emotional processing in the amygdala (Etkin et al. 2011). This regulatory role might have been diminished considering a stressor-induced drop in pgACC activity and heightened scores of perceived fear. Emotion regulation must be relevant for voice production because the amygdala may modulate LMC activity via the SMA, in extremes leading to signs of motor conversion (Boeckle et al. 2016; Simonyan and Fuertinger 2015). Overall, given the deactivation in the thalamus, connected subcortical areas, and the cerebellum, motor programming and motor execution was likely altered, possibly degraded during stress. However, across participants, the final sensorimotor output in primary vocalization areas (i.e., LMC, premotor) was not significantly different between Voice No Stress and Stress conditions.

#### Brain activations for the contrast of voiced versus whispered speech

The contrast of voiced and whispered productions was included to better localize stressor-induced brain changes for vocalization. During the Stress condition, there was less activation in the left STG and more activation in the left precentral gyrus (area 6) during the Whisper than Voice condition. The activation difference included the left dorsolateral LMC in area 6, which is an area that can be recruited for additional indirect control of laryngeal motoneurons in addition to the LMC in area 4p (Simonyan 2014). The greater activation in area 6 for whispered productions during the Stress condition may reflect heightened motor cortical drive to inhibit full laryngeal engagement and posture the glottis in a partially opened state. Increased activation in this region during the Whisper condition may also reflect augmented drive associated with the emotional and cognitive consequences of the stressor condition.

### Correlations of BOLD activations in ROIs with salivary cortisol, personality, and aerodynamic vocal function

The second aim of this study was to determine the neural signature, personality profile, and aerodynamic vocal function in relation to salivary cortisol responses. Hypotheses were partially confirmed.

The hallmark of the Stress condition was a moderate positive correlation of cortisol reactivity with left ACC activation (to a lesser degree L MCC activation). As discussed in the previous section, the ACC and MCC are involved in conflict and action monitoring and heightened activity in stress responders may be linked with processing of emotions and cognitions related to the stressor with consequences for motor activity. The ACC and MCC connect with the SMA via the dlPFC. The (pre)-SMA modulates behavior, and by extension vocal behavior. The pre-SMA, a cognitive-motor area, may energize or inhibit behavior (Wager et al. 2009) while the SMA proper initiates motor behavior (Nachev et al. 2008; Picard and Strick 2001). Only the SMA proper connects directly with the primary motor cortex (Etkin et al. 2011). The pre-SMA is capable of response inhibition or “braking” through “hyperdirect” connections with the subthalamic nucleus and belongs to a motor inhibition network encompassing the right inferior frontal cortex, pre-SMA, globus pallidus, and subthalamic nucleus (Aron et al. 2014). Relatedly, the SMA belongs to a motor conversion disorder network composed of the dlPFC, medial PFC, aMCC, superior frontal gyrus, insula, and amygdala where shifts in prefrontal and cingulate functioning can result in inhibition of motor behavior mediated by a dysfunctional SMA-amygdala connection (Boeckle et al. 2016).

The effect of stressor exposure on LMC activity became clearer once correlations of BOLD signal changes with cortisol data were investigated in addition to comparisons by condition. Cortisol reactivity had a strong negative correlation with L area 4p during Voice No Stress, which decreased to a moderate negative correlation during Voice Stress leading to a stressor-induced increase in L area 4p activity shown in Fig. 6. That is, greater cortisol reactivity was generally linked to lower L area 4p activity. Left area 4p is a critical participant in the initiation and execution of motor commands for speech and the modulation of movement-related attention (Fuertinger et al. 2015). Acute stress did appear to mobilize motor areas for speech production including the LMC in those who were more stress reactive perhaps increasing effort for the initiation and execution of motor commands for speech despite a conflicting communicative situation (wanting to avoid public speaking).

With regard to personality, a key finding was that data converged on a relationship of cortisol reactivity and laryngeal motor activity with extraversion. Greater cortisol reactivity and generally lower L area 4p activity was linked with lower Extraversion-Activity scores across conditions. In addition, lower L area 4p activity correlated with lower airflow during voicing during the No Stress condition. Thus, the data align with the *Trait Theory of Voice Disorders* in that extraversion may play an important role in the relationship between personality and laryngeal behavior, reflecting a less “activated” behavioral state.

During the Stress condition, greater L area 4p activity was linked with greater Conscientiousness-Achievement Striving and greater behavioral inhibition scores, personality attributes that characterized individuals who were less and not more cortisol reactive. During the Stress condition, lower P_sub_ and greater R_law_ correlated with greater L ACC (no correlation with personality) and lower L HC activity (lower Openness-Ideas, Agreeableness-Straightforwardedness), respectively. Perhaps the stressor-induced mobilization of area 4p was partially the result of increased or altered laryngeal airway resistance (subglottal pressure divided by airflow during voicing) to meet the speaking demands during stress. Such an interpretation is supported by a separate study with cortisol data that showed a drop in airflow during voicing with stressor exposure in stress responders but not non-responders, which could be related to a tendency for breathholding (Dietrich, unpublished results). Overall, the stressor-induced neural signature might reflect heightened effort to produce speech in light of insufficient cortical input to initiate speech for at least some participants.

### Limitations and future directions

The study’s focus was on feasibility testing of a stress reactivity protocol coupled with speech production as well as obtaining pilot data. Therefore, the findings are limited by the study’s small sample size. Nonetheless, existing significant findings and trends that underscore differential neural signatures underlying brain responses to stress are promising. Stress responder and non-responder subgroups were small and also imbalanced with only four stress responders although we pre-screened individuals on trait stress reactivity. One reason might be that some participants experienced cortisol reactivity in response to practice in the mock scanner (Fig. 2), which in turn limited the strength of the AUC_I_ formula because it takes into account cortisol levels pre-stressor to identify stress responders. Despite recovery time post practice, only one cortisol data point pre-stressor could be used as baseline, instead of two or three. A practice session on a separate day or longer recovery would be a solution.

There is the potential that a task repetition effect affected the results for the Stress condition. However, the stressor cannot precede the baseline condition. In fact, the pattern of brain activations differed as a function of cortisol reactivity thus supporting the effect of the stress manipulation on the results. The Voice-Whisper contrast may not have adequately isolated laryngeal activity. Whispering also includes laryngeal activity, but is produced with limited vocal fold adduction and no vocal fold vibration. However, interindividual variability in whispering has been recognized and complicates the interpretation of results (Andreatta et al. 2010; Konnai et al. 2017). Further, voice behavioral data were not collected during the MRI session but the accuracy of the tasks was monitored. Future studies should strive to collect acoustic and vocal function data during the MRI session. Finally, functional connectivity analyses will be an important next step to study pathways interconnecting ROIs. BOLD fMRI is inherently limited as the nature of the BOLD signal change in terms of activation or inhibition cannot be distinguished. Coactivation of regions does not equal functional relationships between and among activated regions.

## Conclusion

To the best of our knowledge, this is the first study investigating the central neural effects of a public speaking speech preparation stressor on the phonatory control for speech. The study showed that our fMRI stress reactivity and speech protocol was feasible and that pilot data were promising showing that (a) the key impact of stressor exposure on vocal control occurred in areas considered secondary to phonation control, specifically deactivations in the ACC, MCC, insula, putamen, and thalamus, and (b) there are individual differences in stressor-induced brain activations as a function of stress reactivity, as defined by salivary cortisol, with greater cortisol reactivity linked with lower laryngeal motor cortex activity and lower scores on aspects of extraversion. Our preliminary findings are significant, because they illustrate how perturbations of the speech production system may interfere with the voluntary control of phonation via potential limbic-motor interactions. The individual differences lend preliminary support to key aspects of the *Trait Theory of Voice Disorders* that the limbic system modulates sensorimotor function underlying vocal control differently in those who are more reactive to novelty and threat. However, a link to heightened peripheral laryngeal muscle tension must be studied further. Our data lay the groundwork for future hypothesis-driven research on the effects of stress on voice production in individuals with and without voice disorders such as muscle tension dysphonia. The current findings are critical to interpret disordered processes underlying the central control of voice and psychobiological measures (cortisol, fear, personality). Laryngeal function indices provide important confirmatory correlates to our imaging data.
